# Correction: Yang et al. SOX2 Represses Hepatitis B Virus Replication by Binding to the Viral EnhII/Cp and Inhibiting the Promoter Activation. *Viruses* 2020, *12*, 273

**DOI:** 10.3390/v16121887

**Published:** 2024-12-06

**Authors:** Hua Yang, Jiayin Mo, Qi Xiang, Peiyi Zhao, Yunting Song, Ge Yang, Kailang Wu, Yingle Liu, Weiyong Liu, Jianguo Wu

**Affiliations:** 1 State Key Laboratory of Virology, College of Life Sciences, Wuhan University, Wuhan 430072, China; 2014102040012@whu.edu.cn (H.Y.); mojiayin@whu.edu.cn (J.M.); 2017202040043@whu.edu.cn (Q.X.); 2018202040050@whu.edu.cn (P.Z.); 2018202040016@whu.edu.cn (Y.S.); 2016202040041@whu.edu.cn (G.Y.); wukailang@whu.edu.cn (K.W.); mvlwu@whu.edu.cn (Y.L.); 2 Guangdong Provincial Key Laboratory of Virology, Institute of Medical Microbiology, Jinan University, Guangzhou 510632, China; 3 Department of Clinical Laboratory, Tongji Hospital, Tongji Medical College, Huazhong University of Science and Technology, Wuhan 430030, China

## Error in Figure 2H

In the original publication [1], there was a mistake in “Figure 2H” as published. The image of β-actin in “Figure 2H” was duplicated in “Figure 1B”. Due to an oversight during the manuscript editing process, the manuscript was published with two duplicated images. The corrected “Figure 2H” appears below. The authors state that the scientific conclusions are unaffected. This correction was approved by the Academic Editor. The original publication has also been updated.

## Figures and Tables

**Figure 2:**
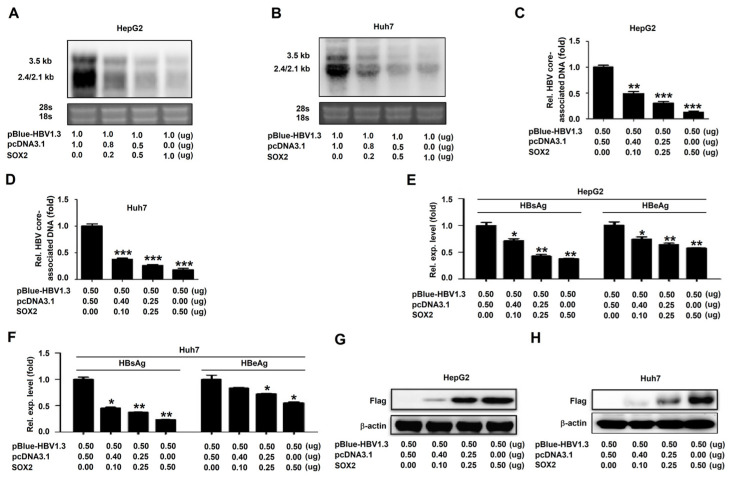
SOX2 represses HBV replication in HepG2 cells and Huh7 cells: (**A**–**H**) HepG2 cells (**A**,**C**,**E**,**G**) and Huh7 cells (**B**,**D**,**F**,**H**) were plated in 6-well plates and then co-transfected with pBlue-HBV1.3 and pcDNA3.1 or pcDNA3.1-SOX2 at different concentrations for 48 h. Total RNA was extracted and HBV RNAs were determined by Northern blot. The 28s and 18s rRNAs were used as the internal controls (**A**,**B**). HBV core-associated DNA was extracted and detected by qRT-PCR (**C**,**D**). HBsAg and HBeAg in cell culture supernatant were analyzed by ELISA (**E**,**F**). SOX2 and β-actin were detected by Western blot (**G**,**H**). *, *p* < 0.05, **, *p* < 0.01 and ***, *p* < 0.001.
